# Resistance of *Escherichia coli* in Turkeys after Therapeutic or Environmental Exposition with Enrofloxacin Depending on Flooring

**DOI:** 10.3390/ijerph15091993

**Published:** 2018-09-13

**Authors:** Bussarakam Chuppava, Birgit Keller, Amr Abd El-Wahab, Jessica Meißner, Manfred Kietzmann, Christian Visscher

**Affiliations:** 1 Institute for Animal Nutrition, University of Veterinary Medicine Hannover, Foundation, Bischofsholer Damm 15, D-30173 Hanover, Germany; bussarakam.chuppava@tiho-hannover.de (B.C.); birgit.keller@tiho-hannover.de (B.K.); 2 Department of Nutrition and Nutritional Deficiency Diseases, Faculty of Veterinary Medicine, Mansoura University, Mansoura 35516, Egypt; amrabdelwahab37@yahoo.de; 3 Institute for Pharmacology, Toxicology, and Pharmacy, University of Veterinary Medicine Hannover, Foundation, Bünteweg 17, D-30559 Hannover, Germany; jessica.meissner@tiho-hannover.de (J.M.); manfred.kietzmann@tiho-hannover.de (M.K.)

**Keywords:** flooring design, Turkey, antibacterial resistance, enrofloxacin, commensal *E. coli*

## Abstract

Gaining knowledge about the spread of resistance against antibacterial agents is a primary challenge in livestock farming. The purpose of this study was to test the effect of double antibiotic treatment (at days 10–14 and days 26–30) with enrofloxacin or solely environmental exposition (identical times, directly into the litter) on resistance against antibacterial agents in commensal *Escherichia coli* in comparison with the control (without treatment), depending on different flooring. A total of 720 Big 6 turkeys participated in three trials. Four different flooring designs were examined: An entire floor pen covered with litter, a floor pen with heating, a partially slatted flooring including 50% littered area, and a fully slatted flooring with a sand bath. A total of 864 *Escherichia coli* isolates were obtained from cloacal swabs and poultry manure samples at days 2, 9, 15, 21, and 35. The broth microdilution method (MIC) was used to determine the resistance of isolates to enrofloxacin and ampicillin. A double antibiotic treatment with enrofloxacin reduced the proportion of susceptible *Escherichia coli* isolates significantly in all flooring designs. Simulation of water losses had no significant effect, nor did the flooring design. Ampicillin-resistant isolates were observed, despite not using ampicillin.

## 1. Introduction

Resistance to antibacterial agents is an increasing problem in public health and veterinary medicine worldwide [1,2,3]. The major public health concern which has been expressed for several decades is still the potential for transmission of antibiotic-resistant bacteria from animals to humans [4]. Most of the amounts of antibiotics used (30–80%) in livestock farming are excreted by the animals directly into the environment via urine and feces because of partial metabolization of antibacterial agents and residue in manure [5,6,7]. Resistance to antibacterial agents in Gram-negative bacteria is on the rise in pathogens as well as in commensal bacterial flora, particularly in *Escherichia coli*. *E. coli* constitutes the majority of invasive Gram-negative isolates for humans in European countries [8]. The natural habitat of *E. coli* is the gastrointestinal tract of mammals and birds [9]. It is considered as an indicator bacteria for resistance detection. *E. coli* also has the ability to survive in and adapt to various extra intestinal habitats and to spread resistances between humans, animals, and the environment [10].

Antibacterial agents in livestock production have been either used to prevent diseases and promote animal growth or for therapeutic purposes [11,12]. The total sales of veterinary antibacterial agents during 2015 in the European Union (EU) amounted to approximately 8361 tons [13]. The average antibacterial consumption by humans (124 mg/kg) was lower than in animals (152 mg/kg) [3]. The resistance level of avian isolates to *E. coli* in Germany, for example, exceeded the level determined by the Federal Office of Consumer Protection and Food Safety for other veterinary pathogens in other animal species [14]. In the past, in relation to their respective fattening periods, in poultry, antibacterial agents have been used more often and for a longer duration compared with cattle and pigs [15].

Fluoroquinolones (FQ) have been classified as being critically important for human health and animal farms by the World Health Organization [4]. An unfavorable situation has arisen in Europe: Resistance to these antibiotics is widespread and the incidence of resistance increased significantly between 2012 and 2015 [3]. The application of FQ agents in poultry husbandry has led to increasing problems with resistance to antibacterial agents [16,17]. The level of fluoroquinolone consumption showed a significant correlation with antibiotic resistance in *E. coli* in livestock husbandry [3,18,19]. In turkeys, commensal and pathogenic *E. coli* are often resistant to quinolones, including enrofloxacin, and to β-lactams [2,13]. Commensal *E. coli* isolates gained from turkey meat in Germany showed higher rates of resistance to FQ than *E. coli* from broilers [20].

In commercial poultry meat production in Europe, turkeys are reared on littered concrete floor. During the fattening period, the primary litter material becomes mixed with poultry excreta, feathers, feed, and spilt drinking water [21], the resulting mixture being referred to as poultry manure. Therefore, close contact with their litter or rather manure is common for turkeys during their productive life. More than 95% of the dry matter in manure consists of excreta [22]. This material can contain residues of antibacterial agents as well as resistant bacteria [17]. On almost every farm (62.3%), *E. coli* can be isolated from manure [23]. The poultry environment has long been acclaimed as a potential source of antibiotic-resistant bacteria [5,17], acting as a possible reservoir for the dissemination of these organisms to humans via the food chain (poultry meat), person-to-person contact (food handlers), and environment (poultry waste disposal, organic fertilizers). A significant proportion of these antibiotics is excreted unchanged in animal urine and feces. These antibiotics can remain potent for a longer time in manure during storage [5,24].

Information concerning the effects of separating animals from their excreta on the development of resistance to antibacterial agents in commensal *E. coli* in rearing turkeys has only been described in the study by Chuppava et al. [25]. The aim of the present study was to evaluate the effect of double antibiotic treatment (at days 10–14 and at days 26–30) with enrofloxacin or solely environmental exposition (at days 10–14 and days 26–30 directly into the litter) on resistance against antibacterial agents in commensal *Escherichia coli* in comparison with the control (without treatment), depending on different flooring. The different types of flooring design were distinguished by means of the contact intensity of birds to their excreta.

## 2. Materials and Methods

The animal experiments were conducted in accordance with the corresponding German regulations and approved by the Ethics Committee of Lower Saxony for Care and Use of Laboratory Animals (LAVES) (Niedersächsisches Landesamt für Verbraucherschutz und Lebensmittelsicherheit; reference: 33.12-42502-04-15/2044).

### 2.1. Design of Experiments

A total of 720 female one-day-old turkeys (B.U.T. Big 6) were obtained from a commercial hatchery (Heidemark GmbH, Ahlhorn, Germany). A total of three independent experiments (T1–T3) were carried out. For each of these experiments, 240 birds were used.

Before starting the experiments in the second week after hatch, the birds were housed in dry and clean floor pens in a quarantine stable. Flooring was covered with wood shavings (GOLDSPAN^®^, Goldspan GmbH and Co. KG, Goldenstedt, Germany). A commercially prepared pelleted diet was offered ad libitum (Best 3 Geflügelernährung GmbH, Twistringen, Germany).

**Figure 1:**
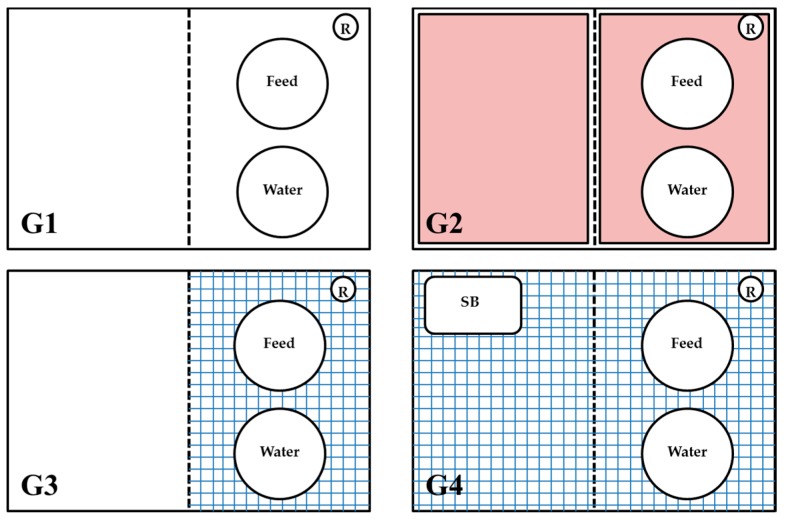
Flooring designs used in the study: **G1** = entire floor pen with litter; **G2** = identical to G1 and additionally having floor heating (in red); **G3** = plastic covered steel slats in 50% of the pen (in blue) as well as an area with litter; **G4** = fully-slatted flooring with plastic covered steel slats and a sand bath (900 cm^2^). SB = sand bath, R = rope.

Each experiment was started after the above described one-week adaptation period. For these experiments, specially manufactured boxes were used, twelve experimental pens (1.20 × 0.80 m) in total. Different flooring designs were used to establish different degrees of contact intensity of the animals to the manure (Figure 1).

The first group served as a control. Animals were kept on dry wood shavings (G1—entire floor pen covered with litter). The second group was identically kept. The exception was an electrical floor heating system (Sauerland GmbH, Paderborn-Elsen, Germany) with an adjuster to control the temperature (G2—floor pen with litter with floor heating). Animals in these two groups continuously had full contact with manure. The pens in the third group (G3) were divided into two equal parts consisting of 50% solid flooring with wood shavings on the right-hand side and 50% plastic slatted flooring on the left-hand side. In the last group (G4), plastic slatted flooring with a sand bath (900 cm^2^) was used, the bath being disinfected and the sand replaced on a daily basis. Animals in G4 had no contact with litter except possibly in the sand bath. Plastic covered steel slats consisted of holes (15 × 10 mm) and bridges (plastic covered steel; 3.5 mm wide; Big Dutchman International GmbH, Vechta, Germany). The excreta were stored under the slatted floor at a depth of approximately 30 cm without any material being removed during the trial, besides small amounts of material needed for the samples as described below.

The boxes were placed in a randomized sequence in blocks of four subgroups (G1–G4) in the same stable, as previously described [25]. Two boxes of each block were placed on the right-hand side and two on the left-hand side of a central corridor (~1.70 m width). Airing was provided by vacuum air ventilation. This system was installed in the ceiling in two rows above the pens. Wood shavings were used as bedding material (1 kg/m^2^). Stocking densities reached a maximum of 25 kg/m^2^. Hanging type feeders were used (Klaus Gritsteinwerk GmbH & Co. KG, Bünde, Germany) as well as bell drinkers (Ferdinand Stükerjürgen GmbH & Co. KG, Rietberg-Varensell, Germany).

Before commencing with the trials one week after hatch, stables and all materials had been disinfected. Also, tests had been performed to exclude the occurrence of Enterobacteriaceae. All birds were allocated to four groups, each with three identical subgroups (n = 20 birds). Rearing was done until day 36. The birds had unlimited access to fresh water and feed (commercial pelleted growing diet). The environmental temperature was gradually reduced from about 33 °C for the one-day-old birds to about 20 °C by day 36. Lights were continuously on between days 1 and 3 and the photoperiod from day 4 onwards amounted to 16 h of light and 8 h of darkness.

In T1 there was no antibiotic treatment. This experiment served as a nontreated control trial. In contrast, animals in T2 were medicated with Baytril^®^ 10% in drinking water (10 mg enrofloxacin/kg body weight per day—corresponding to an addition of 0.5 mL Baytril^®^ 10%/L of drinking water, in accordance with the recommended dosage; Bayer Vital GmbH, Leverkusen, Germany). In the last trial (T3), the birds were not treated with any antibiotic in drinking water. Spillage of drinking water containing enrofloxacin was simulated. The amount of water losses was calculated according to experience from former trials, comparing water intake in turkeys using drinking bowls and nipple drinkers (data not shown). Water containing enrofloxacin (dosage: 0.5 mL/L of Baytril^®^ 10%, amount 240 mL per day) was sprayed into the litter or on the slatted flooring only in the feeding area. Both, in T2 and T3, five-day treatments were performed at days 10–14 and days 26–30.

At the end of day 21, eight out of 20 birds in each subgroup were dissected. Final dissection for all remaining turkeys (n = 12/box) was done at day 36. The stunning method (percussive blow to the head) was conducted in accordance with Annex I of the Council Regulation (EC) No. 1099/2009, Chapter I, Methods, Table 1—Mechanical Methods [26].

### 2.2. Collection of Cloacal Swabs and Manure Samples

Samples (864 in total) were taken before treatment, directly after antibiotic treatment and at the end of the trial. Cloacal swabs were collected at day 2 and manure samples at day 9 before treatment (BT: before treatment stage). After the enrofloxacin treatment (AT), at day 15, manure samples were taken and six days later, cloacal swabs were taken (day 21). Final sampling (ET) was done for both (manure and cloacal swabs) at day 35. Cloacal swabs were always collected from 24 animals per group, i.e., in total, 96 randomly selected animals. Six samples from each type of flooring design (two samples per pen), in total 24 samples of manure, were taken from two defined locations (feeding area and resting area) in every pen for all trial stages (BT, AT, and ET). Manure samples were taken with a plastic cup (6 cm in diameter) which removed the whole litter material at these locations right down to the floor. All samples were immediately transferred to the laboratory for following analyses.

### 2.3. Bacteriological Analyses and E. coli Isolation

The bacteriological investigations were carried out as previously described [25]. In brief: Cloacal swab samples were directly streaked on Gassner agar plates, following an incubation overnight at 37 °C. For manure samples 50 mL of peptone water (Oxoid, Wesel, Germany) as well as the manure sample itself (25 g each) were put into a sterile Whirl-Pak^®^ Bag (Nasco, Fort Atkinson, WI, USA). Bags were mixed for three minutes with a Bag Mixer^®^ 400 VW (Interscience, Saint Nom, France). Using a sterile loop, 10 μL of each mixed-sample was streaked on Gassner agar (Oxoid, Wesel, Germany) and incubated at 37 °C for 18–24 h.

One single blue color colony from each plate was selected and spread onto Columbia blood agar (Oxoid, Wesel, Germany) and Tryptone Bile X-glucuronide (TBX) agar (Oxoid, Wesel, Germany). Incubation was done overnight at 37 °C. Bluegreen colonies on TBX agar detected glucuronidase activity. The positive indole test with Kovac’s indole reagent (Merck, Darmstadt, Germany) was used to confirm the diagnosis.

### 2.4. Antibacterial Susceptibility Testing

The guidelines of the Clinical and Laboratory Standards Institute (CLSI) and the manufacturer’s recommendations were the basis for testing the resistance of *E. coli* isolates using the broth microdilution technique. Micronaut plates (Merlin, Bornheim-Hersel, Germany) with Mueller–Hinton Broth (Merlin, Bornheim-Hersel, Germany) were used to determine minimal inhibitory concentrations (MICs) of enrofloxacin (ENR) and ampicillin (AMP). Dried antibacterial agents in serial dilutions of enrofloxacin and ampicillin were placed in wells of these plates, as previously described by Chuppava et al. [25]. MIC values were determined by visually reading and interpreting the results. As the reference strain, *E. coli* ATCC 25922 was tested concurrently on each testing day.

### 2.5. Screening of Antibacterial Agents in Water Using High Performance Liquid Chromatography

An aliquot of collected water samples with enrofloxacin from days ten to 14 and days 26 to 30 was used for analyses. The concentration of enrofloxacin in the water was determined using high performance liquid chromatography (HPLC) via the method described by Scherz [27]. Exactly 100 μL of the sample was injected into the system with an autosampler (System Gold 508, Beckmann, Munich, Germany). A flow of 1 mL per minute was maintained by the System Gold 126 solvent module (Beckmann, Munich, Germany). A CC250/4 NUCLEODUR 100-5C 18ec (25 cm, Macherey-Nagel, Oensingen, Germany) column was used, this being connected to a precolumn (LiChroCART^®^ 4-4, Li- Chrospher^®^ 100 RP-18e, 5 μm, Merck, Darmstadt, Germany). A fluorescence detector (RF-551 Shimadzu, Nakagyo-ku, Japan) with 280 nm for excitation and 450 nm for emission was used for detection. The mobile phase consisted of 85% citrate buffer pH 3.0 (citric acid monohydrate: 1.80 g/L tri-sodium-citrate-dihydrate: 0.43 g/L). The concentration of enrofloxacin in the water samples was calculated with the external standard method.

### 2.6. Statistical Analyses

The data of resistance to antibacterial agents were performed using the SAS statistical software package version 7.1 (SAS Inst., Cary, NC, USA). MICs were summarized and reported as susceptible (S), intermediate (I), and resistant (R; the results were classified as 1 = S, 2 = I, or 3 = R), where CLSI veterinary breakpoints were available [28]. The analyses were made with these values for the categories. There are no intermediate values between classes one, two, and three. Therefore, a generally high standard deviation has to be tolerated. In the case of completely sensitive isolates at the beginning of the tests, the values are constant at one, i.e. the standard deviation is zero and can therefore not be seen graphically. Significant differences in the means of the resistance results between the four groups of flooring designs were tested using the repeated measures ANOVA (Fisher’s Least Significant Difference (LSD)). This test was also used to determine the differences between the sampling stages and the frequency of resistance between the three trials.

## 3. Results

In total, 864 *E. coli* were isolated and analyzed. These isolates were obtained from 648 cloacal swabs and 216 manure samples at the BT, AT, and ET stages. In the water collected at days ten to 14 and days 26 to 30 in trial 2, the enrofloxacin concentration were 50.17 and 50.62 µg/mL, respectively; in trial 3, water contained 49.87 and 50.42 µg enrofloxacin/mL, respectively.

### 3.1. Differences in Resistance to Antibacterial Agents in E. coli between Sampling Points as Well as between Trials

Enrofloxacin-resistant *E. coli* isolated from cloacal swabs and manure samples were found at the beginning of trial 1 (T1) and showed significantly higher mean resistance rates than in the other trials (Table 1). In contrast, in trials 2 (T2) and 3 (T3), none of the *E. coli* isolates during the BT stage were resistant to enrofloxacin. There were no significant differences between trial 2 and trial 3 during this stage.

Significant differences could be found between the trials during the AT and ET stages (Table 1). Isolates from the cloacal swabs and manure samples from trial 2 showed the significantly highest resistance to enrofloxacin of the isolates after administering Baytril^®^, followed by mean values of trial 1 and trial 3 (cloacal swabs: 2.90, 1.98, and 1.00, respectively; manure samples: 2.63, 2.00, and 1.08, respectively; Table 1). Also, at the ET stage, the results of mean enrofloxacin resistance in trial 2 showed the same relationship to the other experiments (Table 1).

When comparing the sampling stages (Table 1), the means in enrofloxacin resistance were significantly different between trial 1 and trial 2 regarding the *E. coli* isolated from the cloacal swabs (Table 1). For the medicated group (T2), the number of samples with isolation of resistant *E. coli* in materials (cloacal swab and manure sample) significantly increased from the BT to AT stages upon exposure to enrofloxacin. Nevertheless, the *E. coli* from all samples showed no significant differences in the resistance between the AT and ET stages (Table 1).

The results of means in resistance of ampicillin resistant *E. coli* isolates in trials 1, 2, and 3 are presented in Table 2. *E. coli* isolates from cloacal swabs were 100% susceptible to ampicillin during the BT stage except in trial 1. In this trial, isolates showed a significantly higher resistance to ampicillin (G1 = 1.33, G2 = 1.00, G3 = 1.00, respectively; Table 2).

During the AT stage, a significant difference between the three trials occurred in isolates from the cloacal samples. At this point in time, isolates in T3 showed the significantly highest means in enrofloxacin resistance in cloacal swabs, whereas in manure samples, T2-samples had the highest means. During the ET stage in trial 2, the means of ampicillin-resistant *E. coli* isolates from cloacal swabs were significantly higher than in the other trials (Table 2). In manure samples, no more ampicillin resistance was found in T1 during the ET stage.

The results of mean ampicillin resistance in T2 (animals treated twice with enrofloxacin) differed significantly between the sampling days regarding the *E. coli* strains isolated from the cloacal swabs and manure samples. There was a significant increase in means from the BT to the AT stage (Table 2). In trial 3, there was also a significant increase in means of resistance. The percentage of susceptible isolates changed from 100% susceptible isolates to 46% after simulation of water losses with water containing antibiotic. The significance between the AT and ET stages could not be found in all trials (Table 2).

### 3.2. Testing the Effect of Different Flooring Designs on the Resistance to Antibacterial Agents in E. coli

**Figure 2:**
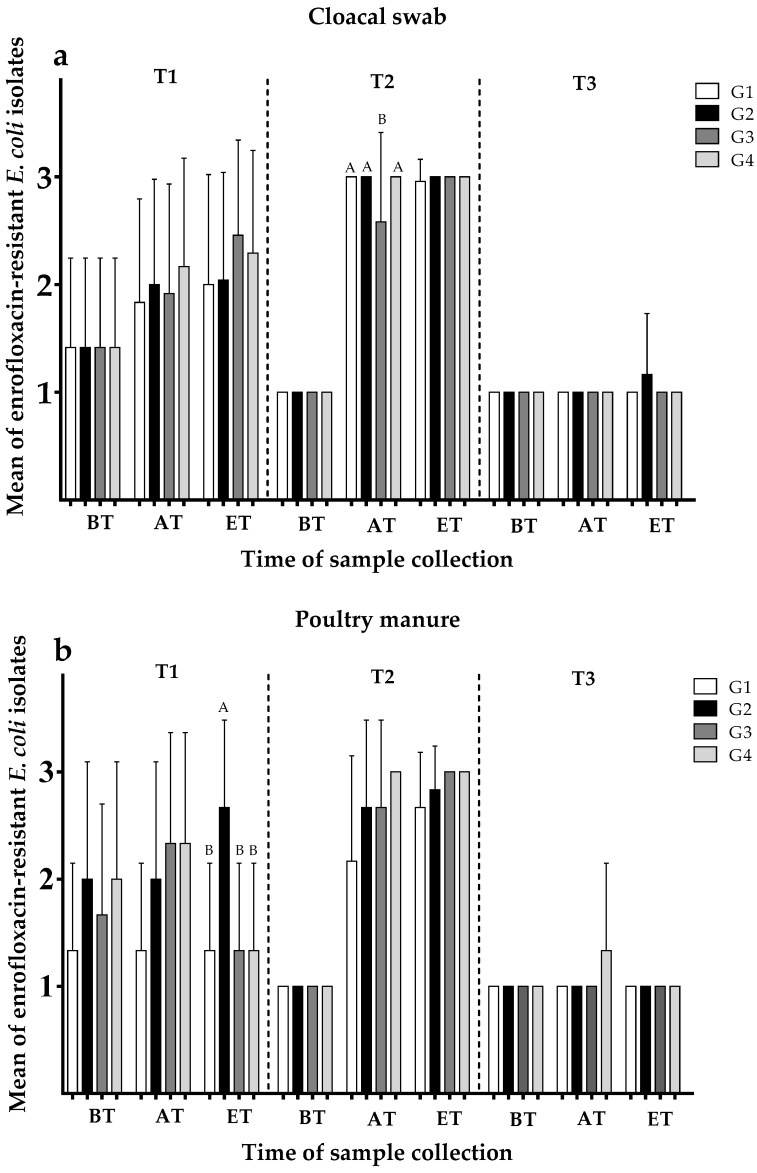

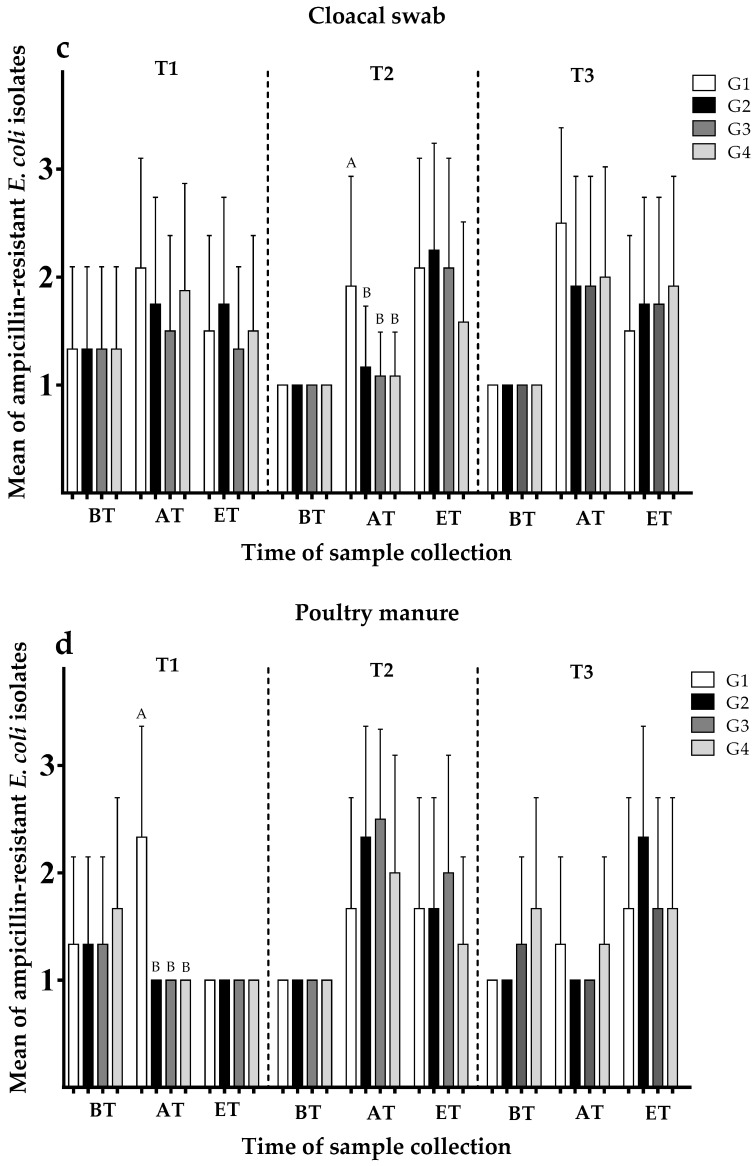
Means of susceptible (=1); intermediate (=2); and resistant (=3) *E. coli* isolates concerning enrofloxacin resistance in (**a**) cloacal swabs and (**b**) poultry manure samples as well as ampicillin resistance in (**c**) cloacal swabs and (**d**) poultry manure samples before treatment (BT), after treatment (AT) and at the end of trial (ET; cloacal swabs: N = 648; per trial BT: n = 24, AT: n = 96, ET: n = 96; poultry manure: N = 216; per trial BT: n = 24, AT: n = 24, ET: n = 24). T1 = no treatment with antibiotic; T2 = treatment of enrofloxacin via drinking water; and T3 = water (containing enrofloxacin) loss simulation trial. G1 = entire floor pen covered with litter; G2 = floor pen covered with litter and having floor heating; G3 = partially (50:50) slatted flooring including an area that was littered; and G4 = fully slatted flooring with a sand bath (900 cm^2^). ^A, B^ means differ significantly between the groups at one sampling (*p* < 0.05).

**Figure 3:**
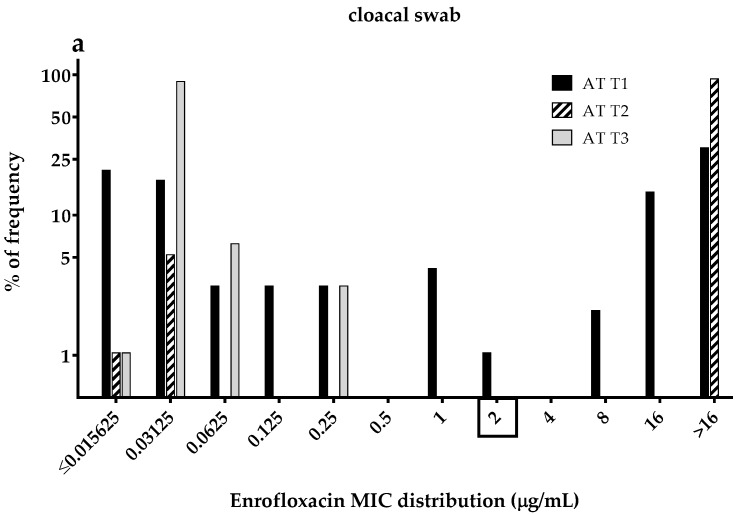

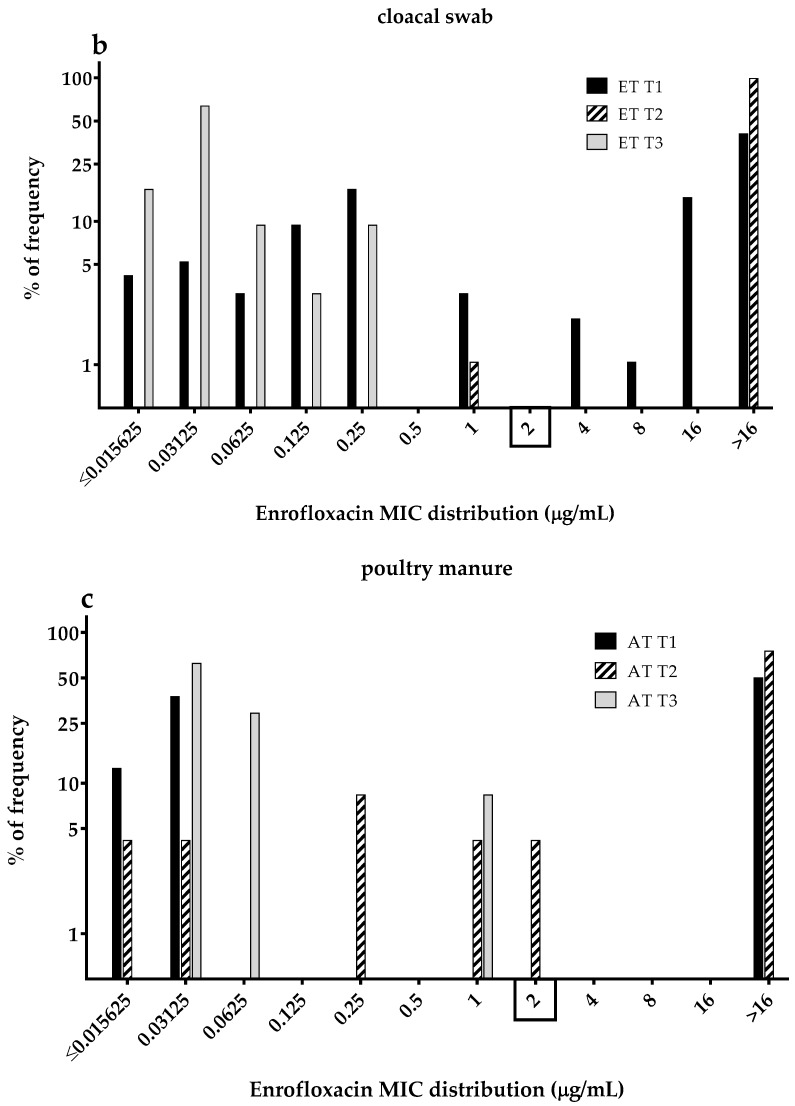

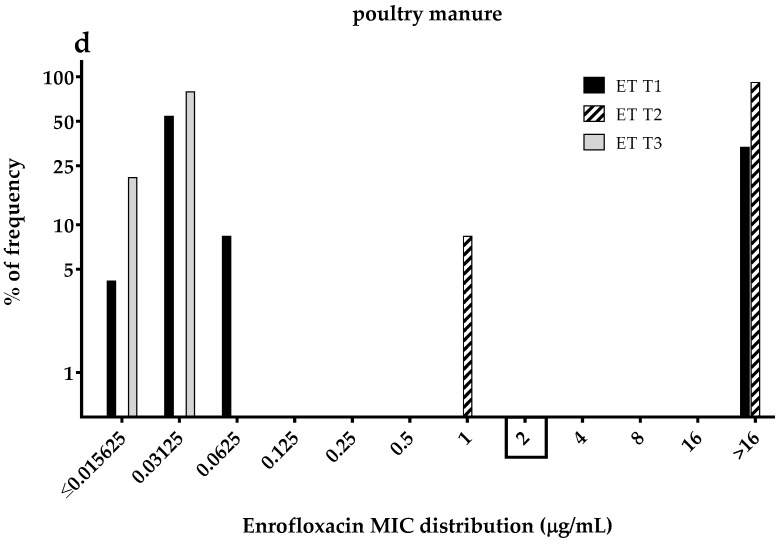
Percentage of frequency of enrofloxacin minimum inhibitory concentration (MIC) distribution in commensal *E. coli* isolates from (**a**) cloacal swabs after treatment (AT) and (**b**) end of trial (ET) as well as in (**c**) poultry manure samples during AT and (**d**) ET of untreated antibiotic (T1), treated twice with enrofloxacin via drinking water (T2) and simulated water spillage with water containing enrofloxacin (T3) in turkeys (cloacal swabs: N = 576; per trial AT: n = 96, ET: n = 96; poultry manure samples: N = 144; per trial AT: n = 24, ET: n = 24). Rectangle on the x-axis: Clinical Laboratory Standard Institute (CLSI) has determined a veterinary specific breakpoint of ≥2 µg/mL enrofloxacin for *E. coli* from chickens and turkeys.

The mean values of resistance of *E. coli* isolates to enrofloxacin and ampicillin depending on sampling stage and flooring design are presented in Figure 2a–d and Figure 3a–d.

#### 3.2.1. Development of Enrofloxacin Resistance Depending on Group

Enrofloxacin resistance in *E. coli* isolates from all samples did not show any differences between the groups during the BT stage. During the AT stage in trial 2 (Figure 2a), *E. coli* isolated from cloacal swabs in G3 showed significantly lower means in resistance rates to enrofloxacin than the isolates collected from animals in other groups showing highest possible means (G3: 2.58; mean values of enrofloxacin resistance in *E. coli* for each group in detail in Supplementary Table S1a). During the ET stage in trial 1, G2 showed significantly higher means in resistance values of enrofloxacin in manure samples (Figure 2b). The *E. coli* isolates in T3 acquired from cloacal swabs and manure samples (Figure 2a,b) were susceptible to enrofloxacin and showed no significant differences between groups; 98% and 96%, respectively.

#### 3.2.2. Development of Ampicillin Resistance Depending on Group

Regarding ampicillin resistance in all trials (Figure 2c,d), there were no significant differences between the groups concerning resistance in isolates from cloacal swabs and manure samples during the BT stages. In contrast, *E. coli* isolates from cloacal swabs during the AT stage in trial 2 (Figure 2c) from G1 demonstrated higher resistance means than in the other groups (G1: 1.92; mean values of ampicillin resistance in *E. coli* for each group in detail in Supplementary Table S1b). In trial 2, the results of means of ampicillin resistance from the manure samples during the AT stage also showed higher values for G1 than observed in either G2, G3, or G4 (2.33, 1.00, 1.00, and 1.00, respectively; Figure 2d; mean values of ampicillin resistance in *E. coli* for each group in detail in Supplementary Table S2b). There was no difference in means of ampicillin-resistant *E. coli* isolated from cloacal swabs and manure samples during the ET stage in all trials (Figure 2c,d).

### 3.3. Enrofloxacin MICs Distributions of the Commensal E. coli Isolates

The percentage of frequency of MICs distribution of the 576 commensal *E. coli* isolates from cloacal swabs and manure samples to enrofloxacin during the AT and ET stages are shown in Figure 3a–d. For *E. coli* from chickens and turkeys, the Clinical Laboratory Standard Institute (CLSI [28]) determined a veterinary specific breakpoint of ≥2 µg/mL for enrofloxacin.

When comparing the three trials (Figure 3a–d), a large number of resistant *E. coli* isolates during the AT and ET stages were found in trial 2, 93% and 99% in cloacal swabs, respectively (Figure 3a,b) and in manure samples, 79% and 92% during the AT and ET stages, respectively (Figure 3c,d). On the other hand, all *E. coli* isolates from trial 3 during the AT stage were susceptible to enrofloxacin (≤0.25 µg/mL).

Regarding the MICs distribution for enrofloxacin, susceptible isolates from cloacal swabs in trial 1 decreased gradually from 52% during the AT stage to 42% at the end of the study (Figure 3a,b). In contrast, the percentage of resistant *E. coli* isolated in trial 2 slightly increased from 93% to 99% during the AT and ET stages, respectively (Figure 3a,b). On the other hand, nearly all of the *E. coli* isolates from the cloacal swabs and manure samples in trial 3 had enrofloxacin MIC-values below the clinical breakpoint (MIC ≤ 2 µg/mL).

## 4. Discussion

The antibacterial agents used in the poultry for treatment or prophylaxis are implicated for the development of bacterial resistance [29]. Treatment in large groups of chickens is often done by oral administration [30]. Some studies reported that antibacterial agents or their metabolites are excreted in manure and residue can therefore be found in the environment [16,31,32].

### 4.1. Consequences of the Oral Administration of Antibacterial Agents

In the present study, one week after the first administration period (d21; AT stage), the amount of resistance was higher compared to the *E. coli* isolates before treatment. A higher rate of enrofloxacin-resistant *E. coli* after oral administration of antibacterial agents was observed in several studies [25,33,34]. Chuppava et al. [25] stated that a single treatment for five days with enrofloxacin led to markedly reduced ratios of susceptible *E. coli* isolates in cloacal swabs and manure samples. The highest proportion of cloacal swabs with resistant *E. coli* was found directly after treatment. Afterwards, a decrease in resistance to enroflaxacin was seen. Therefore, our results are in agreement with those of Chuppava et al. [25] who described a very rapid occurrence of FQ resistance among the commensal *E. coli* after enrofloxacin treatment in poultry. Nevertheless, no difference in resistance in *E. coli* isolates was found between the AT and ET stages after two consecutive treatments with enrofloxacin in this study because MIC values were already very high after one-time treatment.

As expected, after treatment with enrofloxacin, increased MIC values above 2 µg/mL occurred in *E. coli* isolates (T2) with high detection rates up to the end of the trial. Scherz et al. [27] showed that a long-term exposure (21 days) of the commensal flora of poultry to enrofloxacin leads to an amplification and selection of resistant—*E. coli* isolates. These isolates persist in the commensal microbiota. The transmission of *E. coli* isolates of animal origin between the animals in the same pen as well as into the environment may contribute directly to the spread of resistant bacteria in general and may also be a problem for public health [35].

Medication is the main reason for occurrence of resistance to antibacterial agents in *E. coli* [31]. Oral group treatments led to an environmental contamination with antibacterial agents. The application procedure itself or excreted feces from treated animals can be the source [36]. Due to the fact that the metabolic rate of antibiotics is low, 90% of the administered dose is excreted via feces [5]. Avian intestines can act as potential reservoirs of *E. coli* [37]. Thus, there is a higher risk for resistance to antibacterial agents spreading from birds to other birds or from birds to the environment. In other European countries, the higher occurrence of FQ resistance in broilers compared to turkeys has been suggested to depend on an overall use-dependent higher exposure to FQ [3]. It should be noted, however, that the fattening period in turkeys takes much longer under field conditions. Therefore, the resistance situation in the present investigations at the end of the experiment is not comparable with the resistance situation occurring within the normal fattening duration.

In agreement with our data, Jurado et al. [34] and Chuppava et al. [25] found a significant increase in the frequency of resistance to ampicillin in *E. coli* isolates from poultry after orally administering enrofloxacin. These findings may be due to the coselection of β-lactam resistance genes. As the transmissible genetic elements were not analyzed in our study, further studies are recommended in order to confirm the role of such elements in the spread of resistance genes in poultry for *E. coli*.

### 4.2. Effects of the Development of Resistance to Antibacterial Agents in E. coli by Water Loss Simulation (Indirect Administration)

To the best of our knowledge, using water loss simulations by spraying the water containing enrofloxacin exclusively into the litter or onto the slatted flooring in the drinking area in order to study the development of resistance to antibacterial agents has not been previously reported. In this study, it was hypothesized that excreted or metabolized enrofloxacin might alone influence the occurrence of resistance to antibacterial agents. However, in the present study, we could not verify the occurrence of enrofloxacin resistance due to spraying water with enrofloxacin directly into the animals’ environment.

Earlier reports suggested that the carry-over effect of antibacterial agents like FQ as well as their active metabolites in the stable could foster the development of antibacterial resistance via oral ingestion by animals [27]. However, in the present investigation, we sprayed enrofloxacin containing water directly into the environment. In contrast, in the aforementioned study, subtherapeutic dosages (3% and 10% of the recommended dosage of 10 mg/kg body weight) were directly applied to drinking water for 21 days, which could explain the difference. The active dose may therefore have been significantly lower in our own experiments.

Chuppava et al. [25] stated from their experimental model that removing the animals from contaminated pens after antibiotic treatment might be the reason for the lower percentage of resistant *E. coli* isolates in the observed animals. Changing the environment was assumed to lead to a lower percentage of resistant *E. coli* isolates in manure. A lower exposure to resistant bacteria in manure as well as antibacterial agent residues was discussed as the cause for this observation. Additionally, in poultry, dirty or contaminated litter and other animal management parameters affect the microbial composition of the chicken gastrointestinal tract. This influence can be either directly, by providing a continuous source of bacteria, or indirectly, by influencing the physical condition and defence of the birds [37].

### 4.3. Effect of Different Types of Flooring Design on the Development of Resistant E. coli

Up to now, little is known about reducing the development of resistance to antibacterial agents by using different flooring designs simulating varying contact intensity between animals and manure. The development of enrofloxacin and ampicillin resistance in *E. coli* was almost independent of flooring design in the present study. Differences in antibacterial susceptibility of commensal *E. coli* isolates from turkeys depending on flooring design have been previously reported [25]. Chuppava et al. [25] mentioned that flooring design had hardly any effect on the development of resistance against antibacterial agents. Nevertheless, in fully slatted flooring systems, with animals having no contact to their litter, resistance to antibacterial agents still develops in the animals.

In T1, overall, the group with floor heating (G2; average floor temperature in all trials: G1 = 27.0/G2 = 30.5/G3 = 26.5/G4 = 26.0 °C) showed a significantly higher number of resistant *E. coli* isolates than the other groups. Previous studies showed that the resistance to antibacterial agents in animals can change when they are kept in a heat stress environment [25,38]. A high amount of enrofloxacin resistant isolates from cloacal swabs in fattening turkeys was already reported by Chuppava et al. [25] in a group with floor heating. Also, in swine, Moro et al. [38] found a significant increase in resistant *E. coli* isolates in the intestinal flora after the animals had been exposed to heat stress (environmental temperature: 34 °C).

A significantly higher prevalence of ampicillin resistance in *E. coli* isolates from excreta material from cloacal swabs and manure samples was found in the entire floor pen with litter (G1) even when no ampicillin had been administered to the animals and the pens and the stable had been tested and found to be free of *Enterobacteriaceae* at the start of the trial. Further genetic analyses were not conducted. Therefore, the reason for this difference remains unknown.

### 4.4. Natural Resistance to Antibacterial Agents Found in Day-Old Chickens

Turkey poults in this study had not been previously exposed to antibacterial agents. However, *E. coli* was isolated from day-old chicks’ meconium in trial 1. Isolates showed resistance to enrofloxacin (48%) and ampicillin (42%). Similar results were reported in previous studies that found one-day-old chicks to be *E. coli* resistant to enrofloxacin [39] and 100% resistant to ampicillin [25]. It has to be mentioned that also other research groups observed high rates of resistance to antibacterial agents already before treatment as well as in the absence of treatment [40]. A vertical transmission of resistant isolates along the production pyramid can occur [3,41]. Also, contamination in the hatchery environment is possible [42]. Persoons et al. [43] stated that besides management, also hatchery-related factors can influence the occurrence of resistance to antibacterial agents. In newly hatched chicks, the common bacteria in the environment, whether antibacterial susceptible or resistant, colonize the intestines and become part of the intestinal normal microflora. Thus, contamination of chickens via vertical transmission could be a possible explanation for the resistance rates found in our study.

The natural enrofloxacin resistance observed in this present study increased strongly. This increase was higher than after one time treatment, as previously reported [25], despite the absence of antibacterial agent usage (T1). Chuppava et al. [25] suggested, according to their findings, that resistance could be reduced or increased, but not eliminated from the animals even with strict disinfection procedures during the experiment. From literature, it is known that a large number of animals carry resistant *E. coli*. These animals can shed huge numbers of resistant organisms. This could result in a rapid contamination of the other individuals in the same pen and in the stable environment [41]. Resistant bacteria can be ingested by birds from the environment. After entering their gut, these may cause the development of resistant *E. coli.* However, there are several possible mechanisms responsible for the development of quinolone resistance [44].

Therefore, further research is strongly recommended to analyze the genetic basis of resistance in the isolates in order to understand the resistance mechanism`s origin, development and transfer.

## 5. Conclusions

In this study, resistance to enrofloxacin was detected at a very high frequency after treatments with enrofloxacin via drinking water. Therefore, the oral administration of enrofloxacin seems to be associated with a significant increase in the frequency of resistance to enrofloxacin in commensal *E. coli* isolates from turkeys. In addition, prevalence of isolates resistant to ampicillin rose significantly. Resistance to enrofloxacin was not detected when the antibacterial agent substance was indirectly sprayed with water into the environment of fattening turkeys. Flooring structure designs did not directly affect the development of resistance to antibacterial agents, or in groups where the animals had no contact to litter. The existence of resistant *E. coli* isolates in one-day-old birds strongly suggests vertical transmission from parent flocks as one possible explanation.

Furthermore, our results can provide useful information, prompting further studies on quinolone resistance mechanisms in commensal *E. coli* depending on different housing systems. However, we cannot consider all interactions when only one isolate is taken from a sample and then, by way of example, we try to deduce the complexity of the development of resistance. Therefore, research is needed to further investigate possible explanations regarding the mechanism behind the dissemination of enrofloxacin-resistant *E. coli* in fattening turkeys.

## Figures and Tables

**Table 1:** Means of enrofloxacin-resistant *E. coli* isolates from cloacal swab and manure samples from turkeys.

Time of Sample Collection **	Enrofloxacin *					
	Cloacal Swab (N = 648) ***			Manure (N = 216) ***		
	BT	AT	ET	BT	AT	ET
T1	1.42 ^A,b^	1.98 ^B,a^	2.20 ^B,a^	1.75 ^A,a^	2.00 ^B,a^	1.67 ^B,a^
T2	1.00 ^B,b^	2.90 ^A,a^	2.99 ^A,a^	1.00 ^B,b^	2.63 ^A,a^	2.92 ^A,a^
T3	1.00 ^B,a^	1.00 ^C,a^	1.04 ^C,a^	1.00 ^B,a^	1.08 ^C,a^	1.00 ^C,a^

^A, B, C^ means in the same column differ significantly between the experiments (*p* < 0.05); ^a, b^ means differ significantly between the stage of sampling within one experiment (*p* < 0.05); * MICs were summarized and reported as susceptible (S), intermediate (I), and resistant (R). Afterwards the results were classified as 1 = S, 2 = I, or 3 = R and means thereof were calculated; ** BT = before treatment; AT = after treatment; ET = end of trial. T1 = untreated antibiotic trial, T2 = treated antibiotic trial, T3 = trial with simulated water spillage containing antibiotic; *** Cloacal swabs: N = 648; per trial BT: n = 24, AT: n = 96, ET: n = 96; poultry manure: N = 216; per trial BT: n = 24, AT: n = 24, ET: n = 24. G1 = entire floor pen covered with litter; G2 = floor pen covered with litter and having floor heating; G3 = partially (50:50) slatted flooring including an area that was littered; G4 = fully slatted flooring with a sand bath (900 cm^2^).

**Table 2:** Means of ampicillin-resistant *E. coli* isolates from cloacal swab and litter/excreta samples from turkeys.

Time of Sample Collection **	Ampicillin *					
	Cloacal Swab (N = 648) ***			Manure (N = 216) ***		
	BT	AT	ET	BT	AT	ET
T1	1.33 ^A,a^	1.80 ^B,a^	1.52 ^B,a^	1.42 ^A,a^	1.33 ^B,a^	1.00 ^B,a^
T2	1.00 ^B,b^	1.31 ^C,a^	2.00 ^A,a^	1.00 ^B,b^	2.13 ^A,a^	1.67 ^A,a^
T3	1.00 ^B,b^	2.08 ^A,a^	1.73 ^B,a^	1.25 ^AB,a^	1.17 ^B,a^	1.83 ^A,a^

^A, B, C^ means in the same column differ significantly between the experiments (*p* < 0.05); ^a, b^ means differ significantly between the stage of sampling within one experiment (*p* < 0.05); * MICs were summarized and reported as susceptible (S), intermediate (I), and resistant (R). Afterwards the results were classified as 1 = S, 2 = I, or 3 = R and means thereof were calculated; ** BT = before treatment; AT = after treatment; ET = end of trial. T1 = untreated antibiotic trial, T2 = treated antibiotic trial, T3 = trial with simulated water spillage containing antibiotic; *** Cloacal swabs: N = 648; per trial BT: n = 24, AT: n = 96, ET: n = 96; poultry manure: N = 216; per trial BT: n = 24, AT: n = 24, ET: n = 24; G1 = entire floor pen covered with litter; G2 = floor pen covered with litter and having floor heating; G3 = partially (50:50) slatted flooring including an area that was littered; G4 = fully slatted flooring with a sand bath (900 cm^2^).

## Supplementary Materials

The following are available online at http://www.mdpi.com/1660-4601/15/9/1993/s1, Table S1a: Means of enrofloxacin- resistant E. coli isolates from cloacal swab and manure samples from turkeys, Table S1b: Means of ampicillin-resistant E. coli isolates from cloacal swab and manure samples from turkeys.
