# Supplementary material for: Resistance of Escherichia coli in Turkeys after Therapeutic or Environmental Exposition with Enrofloxacin Depending on Flooring
Source: Int J Environ Res Public Health. 2018 Sep 13;15(9):1993. doi: 10.3390/ijerph15091993 (PMC6164043; doi:10.3390/ijerph15091993)
Supplement: Supplementary file 1 [file ijerph-15-01993-s001.pdf]

Supplementary Materials:

Table S1a. Means of enrofloxacin- resistant *E. coli* isolates from cloacal swab and manure samples from turkeys.

Time of sample collection*		Enrofloxacin					
		cloacal swabs (n=24)			manure (n=6)		
		BT	AT	ET	BT	AT	ET
T1	G1	$1.42^{A,b}$	$1.83^{A,a}$	$2.00^{A,a}$	$1.33^{A,a}$	$1.33^{B,a}$	$1.33^{B,a}$
	G2	$1.42^{A,b}$	$2.00^{A,a}$	$2.04^{A,a}$	$2.00^{A,a}$	$2.00^{A,a}$	$2.67^{A,a}$
	G3	$1.42^{A,b}$	$1.92^{A,a}$	$2.46^{A,a}$	$1.67^{A,a}$	$2.33^{A,a}$	$1.33^{B,a}$
	G4	$1.42^{A,b}$	$2.17^{A,a}$	$2.29^{A,a}$	$2.00^{A,a}$	$2.33^{A,a}$	$1.33^{B,a}$
T2	G1	$1.00^{A,b}$	$3.00^{A,a}$	$2.96^{A,a}$	$1.00^{A,b}$	$2.17^{A,a}$	$2.83^{A,a}$
	G2	$1.00^{A,b}$	$3.00^{A,a}$	$3.00^{A,a}$	$1.00^{A,b}$	$2.67^{A,a}$	$2.83^{A,a}$
	G3	$1.00^{A,c}$	$2.58^{B,b}$	$3.00^{A,a}$	$1.00^{A,b}$	$2.67^{A,a}$	$3.00^{A,a}$
	G4	$1.00^{A,b}$	$3.00^{A,a}$	$3.00^{A,a}$	$1.00^{A,b}$	$3.00^{A,a}$	$3.00^{A,a}$
T3	G1	$1.00^{A,a}$	$1.00^{A,a}$	$1.00^{B,a}$	$1.00^{A,a}$	$1.00^{A,a}$	$1.00^{A,a}$
	G2	$1.00^{A,a}$	$1.00^{A,a}$	$1.00^{A,a}$	$1.00^{A,a}$	$1.00^{A,a}$	$1.00^{A,a}$
	G3	$1.00^{A,a}$	$1.00^{A,a}$	$1.00^{B,a}$	$1.00^{A,a}$	$1.00^{A,a}$	$1.00^{A,a}$
	G4	$1.00^{A,a}$	$1.00^{A,a}$	$1.00^{B,a}$	$1.00^{A,a}$	$1.17^{A,a}$	$1.00^{A,a}$

$^{a, b, c}$ means in the same row differ significantly between sampling days at group level ($p < 0.05$);
$^{A, B}$ means in the same column differ significantly between groups on level of sampling day ($p < 0.05$);
*BT = before treatment; AT = after treatment; ET = end of trial;
T1 = untreated antibiotic trial, T2 = treated antibiotic trial, T3 = trial with simulated water spillage containing antibiotic.
G1 = entire floor pen covered with litter; G2 = floor pen covered with litter and having floor heating; G3 = partially (50:50) slatted flooring including an area that was littered; G4 = fully slatted flooring with a sand bath (900 cm^2).

Table S1b. Means of ampicillin-resistant *E. coli* isolates from cloacal swab and manure samples from turkeys.

Time of sample collection*		Ampicillin					
		cloacal swab (n=24)			manure (n=6)		
		BT	AT	ET	BT	AT	ET
T1	G1	$1.33^{A,b}$	$2.08^{A,a}$	$1.50^{A,b}$	$1.33^{A,b}$	$2.33^{A,a}$	$1.00^{A,b}$
	G2	$1.33^{A,a}$	$1.75^{AB,a}$	$1.75^{A,a}$	$1.33^{A,a}$	$1.00^{B,a}$	$1.00^{A,a}$
	G3	$1.33^{A,a}$	$1.50^{B,a}$	$1.33^{A,a}$	$1.33^{A,a}$	$1.00^{B,a}$	$1.00^{A,a}$
	G4	$1.33^{A,a}$	$1.88^{AB,a}$	$1.50^{A,a}$	$1.67^{A,a}$	$1.00^{B,a}$	$1.00^{A,a}$
T2	G1	$1.00^{A,b}$	$1.92^{A,a}$	$2.08^{AB,a}$	$1.00^{A,a}$	$1.67^{A,a}$	$1.67^{A,a}$
	G2	$1.00^{A,b}$	$1.17^{B,b}$	$2.25^{A,a}$	$1.00^{A,b}$	$2.33^{A,a}$	$1.67^{A,ab}$
	G3	$1.00^{A,b}$	$1.08^{B,b}$	$2.08^{AB,a}$	$1.00^{A,b}$	$2.50^{A,a}$	$2.00^{A,a}$
	G4	$1.00^{A,b}$	$1.08^{B,b}$	$1.58^{B,a}$	$1.00^{A,b}$	$2.00^{A,a}$	$1.33^{A,ab}$
T3	G1	$1.00^{A,c}$	$2.5^{A,a}$	$1.50^{A,b}$	$1.00^{A,a}$	$1.33^{A,a}$	$1.67^{A,a}$
	G2	$1.00^{A,b}$	$1.92^{B,a}$	$1.75^{A,a}$	$1.00^{A,b}$	$1.00^{A,b}$	$2.33^{A,a}$
	G3	$1.00^{A,b}$	$1.92^{B,a}$	$1.75^{A,a}$	$1.33^{A,a}$	$1.00^{A,a}$	$1.67^{A,a}$
	G4	$1.00^{A,b}$	$2.00^{AB,a}$	$1.92^{A,a}$	$1.67^{A,a}$	$1.33^{A,a}$	$1.67^{A,a}$

$^{a, b, c}$ means in the same row differ significantly between sampling days at group level ($p < 0.05$);

$^{A, B}$ means in the same column differ significantly between groups on level of sampling day ($p < 0.05$);

*BT = before treatment; AT = after treatment; ET = end of trial;

T1 = untreated antibiotic trial, T2 = treated antibiotic trial, T3 = trial with simulated water spillage containing antibiotic.

G1 = entire floor pen covered with litter; G2 = floor pen covered with litter and having floor heating; G3 = partially (50:50) slatted flooring including an area that was littered; G4 = fully slatted flooring with a sand bath (900 cm^2).
